# Gait Measurement System for the Multi-Target Stepping Task Using a Laser Range Sensor

**DOI:** 10.3390/s150511151

**Published:** 2015-05-13

**Authors:** Ayanori Yorozu, Shu Nishiguchi, Minoru Yamada, Tomoki Aoyama, Toshiki Moriguchi, Masaki Takahashi

**Affiliations:** 1 School of Science for Open and Environmental Systems, Graduate School of Science and Technology, Keio University, 3-14-1 Hiyoshi, Kohoku-ku, Yokohama 223-8522, Japan; 2 Department of Physical Therapy, Human Health Sciences, Graduate School of Medicine, Kyoto University, 53 Kawahara-cho, Shogoin, Sakyo-ku, Kyoto 606-8507, Japan; E-Mails: nishiguchi.shu.82s@st.kyoto-u.ac.jp (S.N.); blue@hs.med.kyoto-u.ac.jp (T.A.); 3 Graduate School of Comprehensive Human Sciences, University of Tsukuba, 3-29-1 Otsuka, Bunkyo-ku, Tokyo 112-0012, Japan; E-Mail: m-yamada@human.tsukuba.ac.jp; 4 Research & Development Division, Murata Machinery, Ltd., 136 Takeda-Mukaishiro-cho, Fushimi-ku, Kyoto 612-8686, Japan; E-Mail: toshiki.moriguchi@drw.muratec.co.jp; 5 Department of System Design Engineering, Keio University, 3-14-1 Hiyoshi, Kohoku-ku, Yokohama 223-8522, Japan; E-Mail: takahashi@sd.keio.ac.jp

**Keywords:** gait measurement, laser range sensor, Kalman filter, data association

## Abstract

For the prevention of falling in the elderly, gait training has been proposed using tasks such as the multi-target stepping task (MTST), in which participants step on assigned colored targets. This study presents a gait measurement system using a laser range sensor for the MTST to evaluate the risk of falling. The system tracks both legs and measures general walking parameters such as stride length and walking speed. Additionally, it judges whether the participant steps on the assigned colored targets and detects cross steps to evaluate cognitive function. However, situations in which one leg is hidden from the sensor or the legs are close occur and are likely to lead to losing track of the legs or false tracking. To solve these problems, we propose a novel leg detection method with five observed leg patterns and global nearest neighbor-based data association with a variable validation region based on the state of each leg. In addition, methods to judge target steps and detect cross steps based on leg trajectory are proposed. From the experimental results with the elderly, it is confirmed that the proposed system can improve leg-tracking performance, judge target steps and detect cross steps with high accuracy.

## 1. Introduction

**Figure 1:**
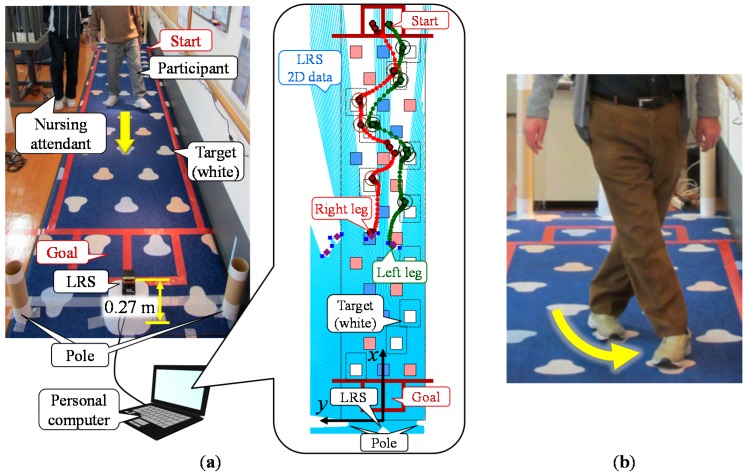
(**a**) An appearance of the multi-target stepping task and proposed gait measurement system; (**b**) Cross step.

Falling is a leading cause of unintentional injury and death in the elderly [1,2] and can also result in impaired mobility, disability, fear of falling and reduced quality of life [3,4,5]. Unsurprisingly, the prevention of falls in the elderly is a public health priority in many countries across the world [6,7,8]. Falling is a common problem in the growing elderly population and there is a need for effective and convenient fall risk assessment tools that can be used in community-based fall prevention programs. Falling occurs in various situations of daily life and generally results from an interaction of multiple and diverse risk factors [1,2,9,10]. Recently, it has been reported that elderly people at high risk of falling show decreases in dual-task performance, *i.e.*, in performing motor and cognitive tasks simultaneously [11,12,13,14]. To prevent falling in the elderly, gait training tasks have been proposed that enhance both motor and cognitive function. One example is the multi-target stepping task (MTST), shown in Figure 1, in which participants step on assigned colored targets arranged randomly on a mat [15]. The MTST evaluates motor function based on the stride length of each leg and the walking speed. Additionally, the MTST judges whether the participant steps on the assigned colored targets (target step judgment) and detects any cross steps (cross step detection) to evaluate cognitive function. The cross step is a behavior where the swinging leg crosses against the supporting leg as shown in Figure 1b. It has been reported that the proportion of missteps on the assigned colored target of high-risk elderly is higher than that of low-risk elderly in the MTST. Moreover, it has been confirmed that cross steps are likely to be seen during a turn when high-risk elderly people perform the MTST [16]. This gait training task requires a gait measurement system to quantitatively measure these parameters for the evaluation of the participant’s dual-task performance capability. To measure these walking parameters and evaluate the risk of falling using the MTST, a measurement system that can measure the foot contact time and position across several meters is required. Furthermore, it is desirable to measure not only the foot contact positions but also the trajectory of both legs during the swing phase.

In many cases, force plates [12,17] or three-dimensional motion measuring devices [18,19] have been used to measure walking parameters such as stride length and walking speed with high reliability. Force plates can assess dynamic balance function and foot contact time and position. However, to measure walking parameters in a several-meter walking test such as the MTST, the measurement system must be configured with several force plates, which is expensive. Three-dimensional motion measuring devices such as the VICON system can capture and analyze the motion of participants with high accuracy. However, the scale of the whole system is larger than the measurement range because of the range of the sensor (IR camera). In addition, it is necessary to attach markers to the participants to capture and analyze their gait. In actual community health centers [20], a non-contact measurement system is desirable because it is necessary to assess many participants in a short time.

In terms of their cost, scale and convenience, it is difficult to install these devices in community health centers. Therefore, since the measurement of the effects of this training is carried out by observation in actual community health centers, it is difficult to quantitatively evaluate the capability of the participants.

To overcome these problems, an ultrasonic sensor, a laser range sensor (LRS) [21] or a RGB-Depth sensor such as the Microsoft Kinect [22] can be used. These devices are comparatively small and inexpensive devices. Several methods of tracking people’s center of gravity using these devices have been proposed [23,24,25,26,27,28,29]. To measure the walking parameters, the system has to track both legs and obtain their positions. A method used to track both legs and measure walking parameters based on the two-dimensional distance data from an LRS has been proposed and verified in straight walking tests [30,31]. Several methods to obtain the posture of a pedestrian based on the RGB-Depth data have also been proposed [32,33,34]. However, in gait training, to avoid the risk of falling for some participants during the MTST, a nursing attendant walks alongside the participant and the participant uses a stick if they use one normally. Additionally, both legs could be close to each other because of a narrow stride, or one leg might be hidden from the sensor owing to the increased number of cross steps in the high-risk elderly. These situations are likely to lead to false tracking or loss of leg tracking entirely. A method to detect and track the legs based on the RGB-Depth data even in cluttered environments has been proposed [35]. To measure walking parameters in several-meter walking tests such as the MTST, the sensor must be able to obtain high accuracy distance data over a wide range. Moreover, to assess the fall risk of elderly people during the MTST, methods to judge target steps and detect cross steps are required.

In this study, we develop a gait measurement system using a laser range sensor (LRS) [21] as shown in Figure 1a. The LRS is a comparatively small and inexpensive device and can obtain high accuracy two-dimensional distance data over a wide range. To reduce the number of occurrences of lost tracking of legs and of false tracking, we propose a novel leg detection method with five observed leg patterns and global nearest neighbor (GNN)-based [36,37] data association with a variable validation region based on the state of each leg. In addition, we propose methods to judge target steps and detect cross steps based on the trajectory of the legs. Comparing the experimental results of the MTST with the video analysis, we confirmed that the proposed system can improve leg-tracking performance in the elderly, judge target steps and detect cross steps. We also confirmed the validity of walking parameters such as foot contact time and position obtained by the proposed system from the results of the target step judgment.

## 2. Gait Measurement System

### 2.1. Configuration

As shown in Figure 1a, the system consists of an LRS, a personal computer, and two calibration poles. In the system, the LRS is installed at shin height (0.27 m in our system) and captures distance data by scanning a single laser beam in a horizontal plane. The personal computer acquires data from the LRS and calculates the leg positions.

### 2.2. Algorithm

**Figure 2:**
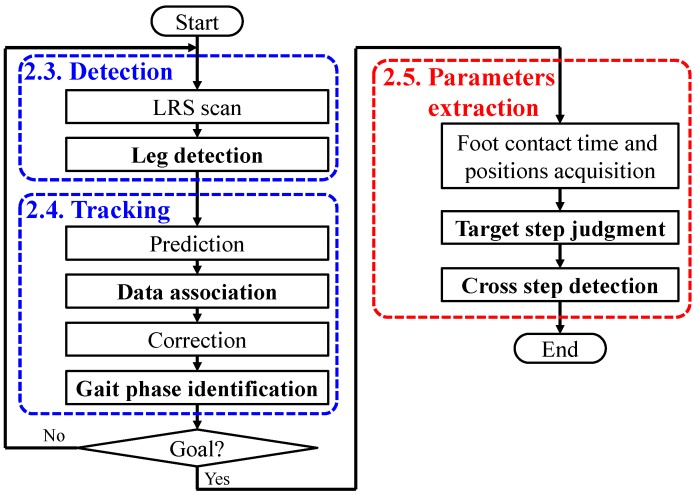
Algorithm of the gait measurement system using an LRS.

As shown in Figure 2, the system has two main processes. The first process is leg detection and tracking. The positions of the legs are calculated based on the proposed leg patterns from LRS scan data. In the proposed system, tracking of the legs is carried out based on a Kalman filter. In addition, the data association (one-to-one matching of a tracked leg and an observed position with an LRS) has been implemented for reliable tracking [37]. In the data association, a validation region is used for eliminating unlikely observation-to-track associations [23]. A validation region is constructed around the predicted position. In this study, GNN-based [36,37] data association with a variable validation region based on the state of each leg is proposed. The second process is extraction of the walking performance parameters of the MTST (foot contact time and position, target step judgment and cross step detection) based on the trajectory of the legs.

**Figure 3:**
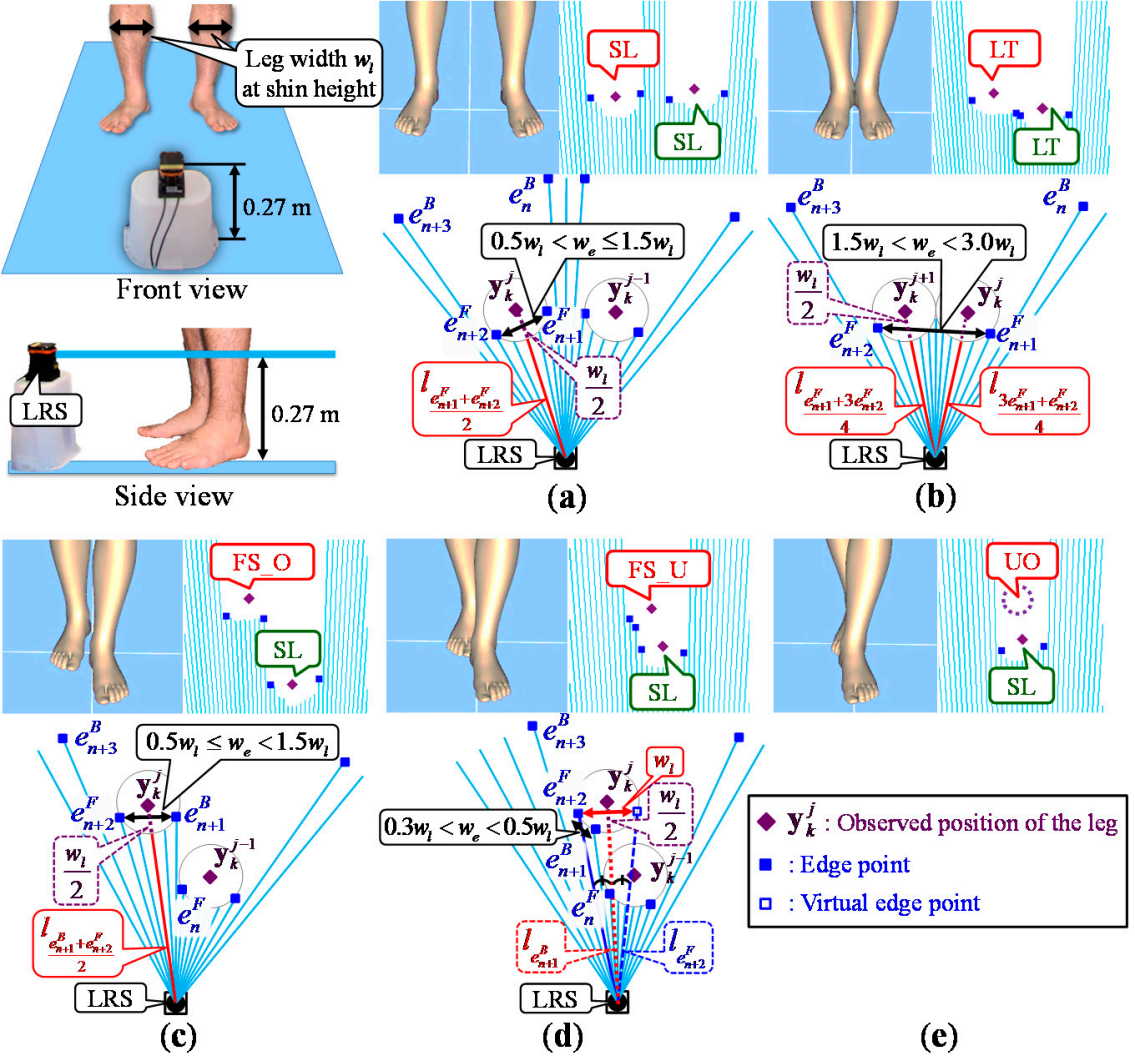
Leg detection using five observed leg patterns; (**a**) SL pattern; (**b**) LT pattern; (**c**) FS_O pattern; (**d**) FS_U pattern; (**e**) UO pattern.

Before walking measurement, the system measures the leg width *w_l_* of the participant at shin height shown in Figure 3 and aligns the mat and LRS using two poles in reference [38].

### 2.3. Leg Detection

This study presents a novel leg detection method to calculate observed leg positions based on the leg width *w_l_* and five observed leg patterns. To calculate the leg positions, the system searches for edges $e_{m}^{h}$ (*m* = 1, …, *M_k_*) from the LRS scan data using the following equation:

(1) $$\left|l_{i}-l_{i+1}\right|>w_{l}/2$$

where *l_i_* is the *i*-th laser-scanned distance data from the right of an LRS. Moreover, the detected edges are identified by $e_{m}^{B}$ = *i*, $e_{m+1}^{F}$ = *i* + 1 when *l_i_* > *l_i_*_+1_, and $e_{m}^{F}$ = *i*, $e_{m+1}^{B}$ = *i* + 1 when *l_i_* < *l_i_*_+1_ (*h* = *F*, *B*, where *F* and *B* indicate the forward and backward edges, respectively). *M_k_* is the total number of detected edges at time step *k*. As shown in Figure 3, the system calculates the observed leg positions $y_{k}^{i}$(*j* = 1, …, *J*) considering five observed leg patterns based on their spatial relationship and the width *w_e_* between the edges. The five observed leg patterns are SL (Single Leg), LT (Legs Together), FS_O (Forward Straddle Observable), FS_U (Forward Straddle Unobservable) and UO (Unobservable).

SL is a pattern in which one leg is fully observable by the sensor alone, and is detected as a sequence of edges {$e_{n}^{B}$, $e_{n+1}^{F}$, $e_{n+2}^{F}$, $e_{n+3}^{B}$}, with a width condition of 0.2*w_l_* < *w_e_* ≤ 1.5*w_l_*. As shown in Figure 3a, the observed position of the leg is calculated based on *w_l_*.

LT is a pattern in which two legs are fully observable side by side by the sensor, and are detected as a sequence of edges {$e_{n}^{B}$, $e_{n+1}^{F}$, $e_{n+2}^{F}$, $e_{n+3}^{B}$} or {$e_{n}^{F}$, $e_{n+1}^{B}$, $e_{n+2}^{F}$, $e_{n+3}^{B}$} or {$e_{n}^{B}$, $e_{n+1}^{F}$, $e_{n+2}^{B}$, $e_{n+3}^{F}$}, with a width condition of 1.5*w_l_* < *w_e_* < 3.0*w_l_*. As shown in Figure 3b, the observed positions are calculated assuming that those two legs are side by side.

FS_O is a pattern in which one leg is observed as a stepped shape by the sensor owing to the influence of the other leg or a stick, and is detected as a sequence of edges {$e_{n}^{F}$, $e_{n+1}^{B}$, $e_{n+2}^{F}$, $e_{n+3}^{B}$} or {$e_{n}^{B}$, $e_{n+1}^{F}$, $e_{n+2}^{B}$, $e_{n+3}^{F}$}, with a width condition of 0.5*w_l_* ≤ *w_e_* < 1.5*w_l_*. As shown in Figure 3c, the observed position is calculated in the same way as in the SL pattern.

FS_U is a pattern that has a similar situation to FS_O, where the leg is again detected as a sequence of edges {$e_{n}^{F}$, $e_{n+1}^{B}$, $e_{n+2}^{F}$, $e_{n+3}^{B}$} or {$e_{n}^{B}$, $e_{n+1}^{F}$, $e_{n+2}^{B}$, $e_{n+3}^{F}$}, with a width condition 0.2*w_l_* < *w_e_* < 0.5*w_l_*. However, the position of the leg cannot be directly calculated. Thus, as shown in Figure 3d, the observed position is calculated virtually based on the leg width *w_l_*.

UO is a pattern in which one leg is unobservable because of occlusion. In particular, even if the leg is not fully observable by the sensor, by calculating the position of the tracked leg in the FS_U pattern, improvements of the estimation accuracy and tracking performance can be expected.

### 2.4. Leg Tracking

This study presents a novel leg tracking method using a Kalman filter and GNN-based data association with a variable validation region based on the state of each leg. If the sampling time ∆*t* (0.05 s in our system) is sufficiently shorter than the gait cycle time, we assume that the change in velocity at the next time step is not very large. The discrete time model of leg motion is given as follows:

(2) $$x_{k}^{f}=\text{A}{\text{x}}_{k-1}^{f}+\text{B}\Delta {\text{x}}_{k-1}^{f}\;\left(f=L,\;R\right)$$

where $\text{A}=\left[\begin{array}{cccc} 1 & 0 & \Delta t & 0\\ 0 & 1 & 0 & \Delta t\\ 0 & 0 & 1 & 0\\ 0 & 0 & 0 & 1 \end{array}\right],\text{ B}=\left[\begin{array}{cc} \Delta t^{2}/2 & 0\\ 0 & \Delta t^{2}/2\\ \Delta t & 0\\ 0 & \Delta t \end{array}\right]$, and $x_{k}^{f}={\left[\begin{array}{cccc} x_{k}^{f} & y_{k}^{f} & {\dot{x}}_{k}^{f} & {\dot{y}}_{k}^{f} \end{array}\right]}^{T}$. $\left(x_{k}^{f},\;y_{k}^{f}\right):={\text{p}}_{k}^{f}$ is the estimated position and $\left({\dot{x}}_{k}^{f},\;{\dot{y}}_{k}^{f}\right):={\text{v}}_{k}^{f}$ is the estimated velocity of the leg (*f* = *L*, *R*, where *L* and *R* indicate the left and right legs, respectively). $\Delta {\text{x}}_{k}^{f}={\left[\begin{array}{cc} n^{{\dot{x}}_{k}} & n^{{\dot{y}}_{k}} \end{array}\right]}^{T}$ is the acceleration disturbance vector, which is assumed to be zero mean and has a white noise sequence with variance **Q**. We set the variance as $\text{Q}=\text{diag}\left[{\left(5.0\right)}^{2}\;,\;{\left(5.0\right)}^{2}\;\right]$ considering that the leg speed is accelerated and decelerated 0.0 to 2.5 m/s in the swing phase (about 1.0 s) in the experiments. The LRS obtains the leg position from $y_{k}^{f}={\left[\begin{array}{cc} x_{k}^{f} & y_{k}^{f} \end{array}\right]}^{T}$. The measurement model is as follows:

(3) $$y_{k}^{f}=\text{C}{\text{x}}_{k}^{f}+{\text{w}}_{k}$$

where $\text{C}=\left[\begin{array}{cccc} 1 & 0 & 0 & 0\\ 0 & 1 & 0 & 0 \end{array}\right]$. ${\text{w}}_{k}={\left[\begin{array}{cc} n^{x_{k}} & n^{y_{k}} \end{array}\right]}^{T}$ is the measurement noise, which is assumed to be zero mean and has a white noise sequence with variance **R**. In our experiments, we set the variance as $\text{R}=\text{diag}\left[{\left(w_{l}/2\right)}^{2}\;,\;{\left(w_{l}/2\right)}^{2}\right]$ considering that the LRS measures the distance within the error and the observed leg position is calculated from the leg width *w_l_*.

#### 2.4.1. Prediction

**Figure 4:**
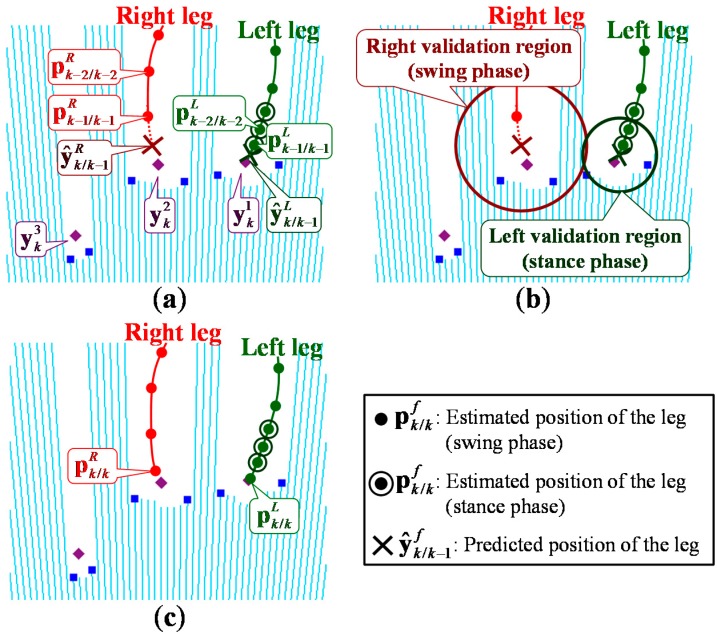
Leg tracking using validation regions considering the state of each leg; (**a**) Prediction; (**b**) Data association; (**c**) Correction.

As shown in Figure 4a, based on the model of leg motion, the system predicts the position of the tracked leg by:

$${\hat{y}}_{k/k-1}^{f}=\text{C}{\hat{x}}_{k/k-1}^{f}=\text{CA}{\hat{x}}_{k-1/k-1}^{f}$$

where ${\hat{\text{x }}}_{k/k-1}^{f}$ and ${\hat{\text{x }}}_{k-1/k-1}^{f}$ are the a priori state estimation at time step *k* and the a posteriori state estimation at time step k−1.

#### 2.4.2. Data Association

As shown in Figure 4b, a validation region is constructed around the predicted position to eliminate unlikely observation-to-track associations. The *j*-th (*j* = 1, …, *J*) observed position $y_{k}^{j}$ is included in the validation region of the predicted position ${\hat{y}}_{k/k-1}^{f}$ of the tracked leg according to:

(5) $$\left\|y_{k}^{j}-{\hat{y}}_{k/k-1}^{f}\right\|<r_{val}^{f}$$

where $r_{val}^{f}$(*f* = *L*, *R*) is the radius of the validation region. The measurement accuracy changes in accordance with the velocity of the leg and whether the leg is moving while hidden. In these situations, losing track of the leg or false tracking of another leg or a stick is likely to occur. To solve these problems, the radius of the validation region is designed considering the state of each leg: gait phase (whether the leg is in the stance phase or swing phase), the speed, and times when the leg is unobservable. The radius of validation region considering these points is shown in Table 1. $H_{k}^{f}$ is the number of times that no observed positions are included in the validation region (observed leg pattern is UO) continuously at time step *k*. *v_sw_* and *v_st_* are the assumed speed of the leg in the swing and stance phases while it is hidden. In our experiments, *v_sw_* and *v_st_* are respectively set to 1.1 m/s and 0.55 m/s considering that the average human walking speed is about 1.1 m/s.

**Table 1:** Setting of the radius of validation region $r_{val}^{f}$ considering the state of each leg.

Gait phase at *k* − 1	Observable	Unobservable
Stance phase	$\frac{3}{4}w_{l}+\Delta t\;\left\|{\text{v}}_{k-1}^{f}\right\|$	$\frac{3}{4}w_{l}+\Delta t\;H_{k}^{f}\;v_{st}$
Swing phase	$w_{l}+\Delta t\;\left\|{\text{v}}_{k-1}^{f}\right\|$	$w_{l}+\Delta t\;\left\|{\text{v}}_{k-1}^{f}\right\|+\Delta t\;H_{k}^{f}\;v_{sw}$

Then, the following cost matrix **D** is defined for observation-to-track associations:

(6) $$D=\left[\begin{array}{cccc} d_{L,1} & d_{L,2} & \cdots & d_{L,J}\\ d_{R,1} & d_{R,2} & \cdots & d_{R,J} \end{array}\right]$$

The element $d_{f,j}$ of the cost matrix is the matching cost between the predicted position ${\hat{y}}_{k/k-1}^{f}$ of the tracked leg and *j*-th observed position $y_{i}^{k}$ and has the following values:

(7) $$d_{f,j}=\{\begin{array}{cc} \lambda_{f,j} & \text{ if }y_{k}^{j}\text{ is in the validation region of }{\hat{y}}_{k/k-1}^{f}\\ \infty & \;\mathrm{else} \end{array}$$

$\lambda_{f,j}$ is the Mahalanobis distance and is calculated as follows:

(8) $$\lambda_{f,j}=\sqrt{{\left(y_{k}^{j}-{\hat{y}}_{k/k-1}^{f}\right)}^{T}{\left(S_{k}^{f}\right)}^{-1}\left(y_{k}^{j}-{\hat{y}}_{k/k-1}^{f}\right)}$$

where $S_{k}^{f}$ is the covariance of the innovation $\left(y_{k}^{j}-{\hat{y}}_{k/k-1}^{f}\right)$. The data association is achieved so that the summed total distance of **D** can be minimized [36].

#### 2.4.3. Correction

Finally, as shown in Figure 4c, based on the result of the data association, the state estimation vector is updated using the Kalman filter. If there are no corresponding observed positions in the validation region, the predicted position ${\hat{y}}_{k/k-1}^{f}$ is used as an observed position and the observed leg pattern is assumed to be UO.

#### 2.4.4. Gait Phase Identification

From validation compared with a force plate [38], it is possible to identify the phase of gait (stance phase or swing phase) considering the speed of both legs in human walking. The condition that the right leg is in the stance phase is:

(9) $$\left\|v_{k}^{R}\right\|<\left\|v_{k}^{L}\right\|\;\vee \;\left\|v_{k}^{R}\right\|<v_{st\_th}$$

The condition that the right leg is in the swing phase is:

(10) $$\left\|v_{k}^{R}\right\|>\left\|v_{k}^{L}\right\|\;\vee \;\left\|v_{k}^{R}\right\|>v_{sw\_th}$$

where $v_{st\_th}$ and $v_{sw\_th}$ are the thresholds of the maximum speed in the stance phase and the minimum speed in the swing phase, respectively. In our experiments, $v_{st\_th}$ and $v_{sw\_th}$ are respectively set to one sixth (0.18 m/s) and one third (0.37 m/s) of the average human walking speed (1.1 m/s). The gait phase of the left leg is identified in the same way.

With the proposed data association method, we can expect that the chances of losing a tracked leg will be reduced even if the velocity of the leg changes suddenly. We can also expect that the chances of false tracking of other observed objects such as another leg or a stick will be reduced because it is difficult for other objects to be included. In addition, the variable validation region is also effective even when the leg is moving while hidden from the sensor.

### 2.5. Walking Parameters Extraction

#### 2.5.1. Foot Contact Position Extraction

**Figure 5:**
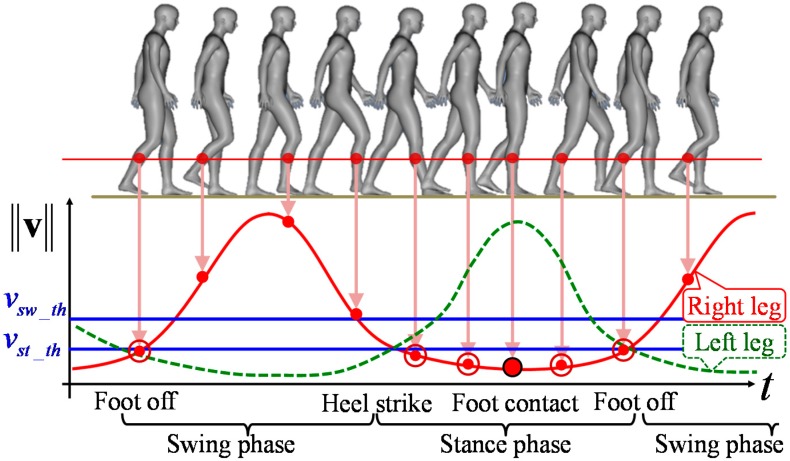
Image of the gait speed diagram during walking.

In this study, the foot contact time is defined as the time when the bottom of the foot is attached to the floor and the leg is perpendicular to the floor. As shown in Figure 5, the speed of the leg at shin height scanned by LRS is at a minimum value during the stance phase. Therefore, the foot contact time is extracted as the time when the leg speed is at a minimum value in the stance phase. In addition, the foot contact position can be acquired as the estimated position at shin height at the foot contact time because the leg is almost perpendicular to the floor.

#### 2.5.2. Target Step Judgment

**Figure 6:**
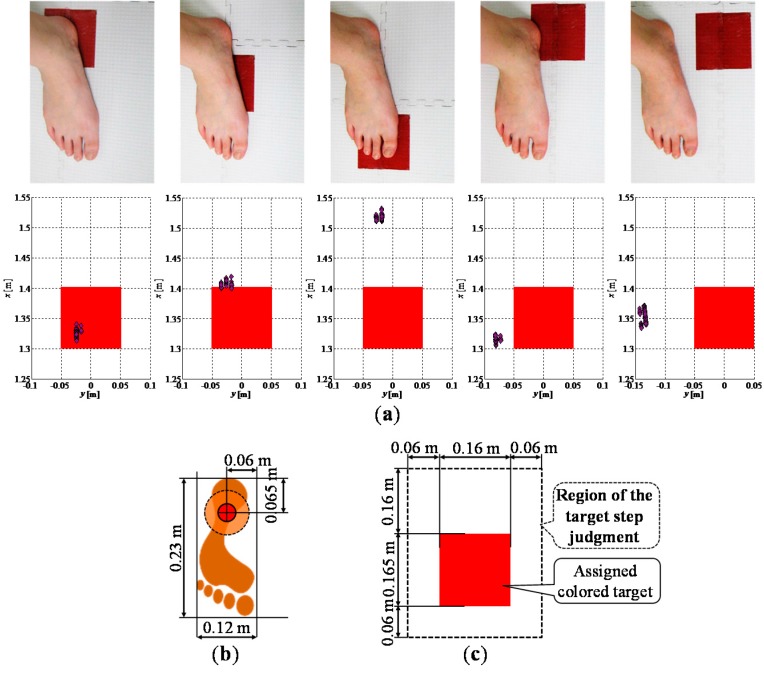
Target step judgment; (**a**) Examples of the results of observed leg position; (**b**) Leg model; (**c**) Region of the target step judgment.

Figure 6a shows the examples of the observed leg position when the participant stepped around the target (target size is 0.160 m × 0.165 m). From the experimental results and the leg model based on the average value of the physical data shown in Figure 6b, to judge whether the participant stepped on the assigned target, the region of the target step judgment was designed as shown in Figure 6c. The system judged that the participant stepped on the assigned colored target if the foot contact position was included in this region.

#### 2.5.3. Cross Step Detection

**Figure 7:**
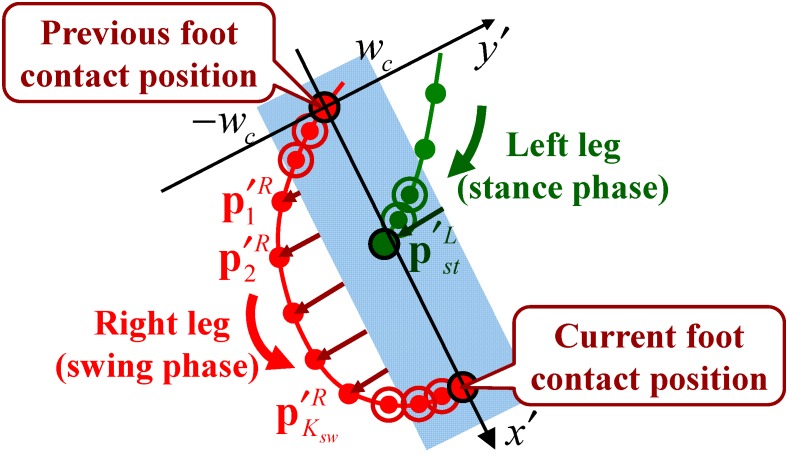
Cross step detection.

As shown in Figure 7, from preliminary experimental data with the elderly, the characteristic relationship between the trajectory of the swinging leg and the foot contact position of the supporting leg was confirmed when the participant performed a cross step. This study presents a method of detecting cross steps based on this relationship.

As shown in Figure 7, an $x^{\prime }-y^{\prime }$ coordinate system whose origin was the previous foot contact position of the swinging leg (right leg in this case) was defined. In this coordinate system, the foot contact position of the supporting leg (left leg in this case) was defined as ${p^{\prime }}_{\mathrm{st}}^{L}={\left[{x^{\prime }}^{L}\;{y^{\prime }}^{L}\right]}^{T}$, and the $k_{sw}\left(=1,\cdot \cdot \cdot ,K_{sw}\right)$-th position from the previous foot contact position of the swinging leg was ${p^{\prime }}_{k_{sw}}^{R}={\left[{x^{\prime }}_{k_{sw}}^{R}\;{y^{\prime }}_{k_{sw}}^{R}\right]}^{T}$. $K_{sw}$ indicates the number of samples in the swing phase. Then, the system detected a cross step if these parameters satisfied the following condition:

(11) $${y^{\prime }}^{L}<w_{c}\;\wedge \;\exists {y^{\prime }}_{k_{sw}}^{R}<-w_{c}\;\left(k_{sw}=1,\cdot \cdot \cdot ,K_{sw}\right)$$

where *w_c_* is the threshold of cross step detection. We determined that *w_c_* = *w_l_*/2 from the experimental results. Cross step detection was performed for the left leg in the same way. The system performed the above processing in every foot contact position and recorded the number and position of the detected cross steps.

## 3. Experiments

### 3.1. Participants and Environment

Sixteen elderly volunteers (eleven men, five women, mean age 78.1 ± 8.7 years), including two elderly people using a stick, were recruited as participants for this study. None of them had any indications of the following symptoms: serious visual impairment, inability to ambulate independently, symptomatic cardiovascular disease, or severe arthritis. Informed consent was obtained from all volunteers prior to participation, in accordance with the guidelines approved by the Kyoto University Graduate School of Medicine (approval number E-880) and the Declaration of Human Rights, Helsinki, 1975.

Table 2 shows the specification of the LRS (UTM-30LX, Hokuyo Automatic Co., Ltd., Osaka, Japan [21]). The sampling time of the system ∆*t* was set to 0.05 s. The MTST mat size was 5.85 m long by 1.15 m wide, and three colored (red, blue and white) targets (0.160 m × 0.165 m) were arranged randomly on it. As shown in Figure 1a, participants walked from the start position to the goal position stepping on the assigned colored targets three times (three colors). To avoid the risk of falling during the MTST, a nursing attendant walked alongside the participant.

**Table 2:** Specifications of the UTM-30LX LRS ([21]).

Detection Range	0.1–30 m, max. 60 m
	270°
Measurement Accuracy	0.1–10 m: ±0.03 m
	10–30 m: ±0.05 m
Angular Resolution	0.25°(360°/1440)

### 3.2. Verification of Leg Tracking

To verify the effectiveness of the proposed leg tracking method, three conventional methods labelled 1 to 3 (see Table 3 for definitions) were used. In Method 1, conventional leg detection method excluding the FS_U pattern [27] was used. In Methods 2 and 3, the proposed leg detection method using the FS_U pattern was used. We set a large fixed validation region for each method considering the observation error and the moving distance at one sampling time point in the swing phase without prediction. In Methods 1 and 2, a radius of the large fixed validation region $\left(r_{val}^{f}=\frac{3}{2}w_{l}\right)$ was used. The average leg width *w_l_* at shin height is about 0.1 m. We assumed that the observation error was *w_l_*/2 and that the moving distance was *w_l_* considering that the leg speed in the swing phase was twice the average human walking speed (1.1 m/s) and that the sampling time was 0.05 s in this system. In Method 3, a radius of the small fixed validation region ($r_{val}^{f}$ = *w_l_*) was used. We assumed that that the observation error was *w_l_*/2 and the moving distance was *w_l_*/2 considering that the leg speed in the stance phase was the same as the average human walking speed.

**Figure 8:**
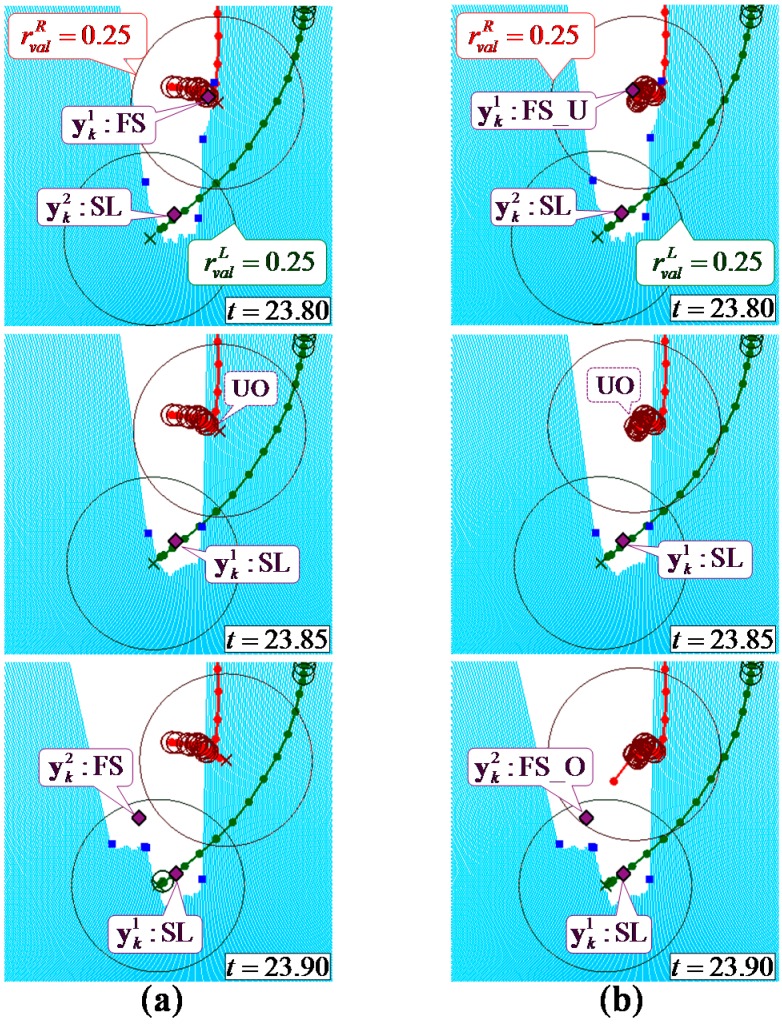
Example of leg tracking results in a situation where the right leg of the participant was temporarily hidden; (**a**) Method 1: conventional leg detection excluding the FS_U pattern; (**b**) Method 2: the proposed leg detection using the FS_U pattern.

**Figure 9:**
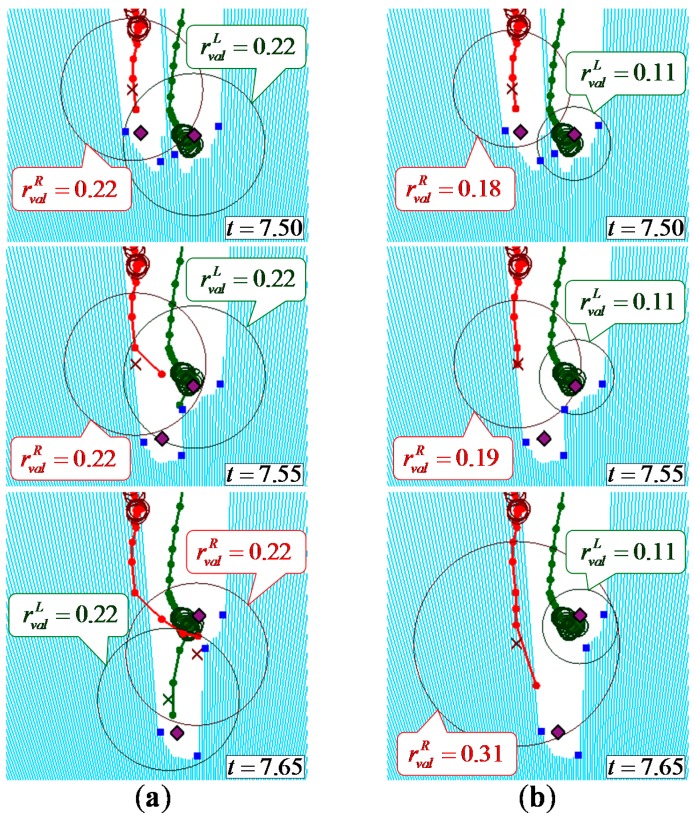
Example of leg tracking results in a situation where both of the participant’s legs were close together; (**a**) Method 2: the radius of the large fixed validation region was used; (**b**) Proposed method: the radius of the validation region was changed depending on the state of each leg.

Figure 8 and Figure 9 show example leg-tracking results in those situations that are likely to lead to false tracking or losing track of the legs. In addition, Table 3 shows all 48 gait measurement results of 16 elderly people walking.

Figure 8 shows an example of the LRS data and gait measurement results in a situation where the right leg was temporarily hidden. As shown in Figure 8, the right leg was hidden by the left leg at time *t* = 23.80. In Method 1, which excluded the FS_U pattern for leg detection, the estimated position deviated significantly at time *t* = 23.85 because an accurate observed position could not be obtained at time *t* = 23.80. Therefore, the system lost track of the right leg. In Method 2, used the FS_U pattern for leg detection, even if the leg was not fully observable at time *t* = 23.80, by calculating the position of the tracked leg in the FS_U pattern, the system could obtain an accurate estimated position at time *t* = 23.85. The system could therefore keep track of the right leg.

Figure 9 shows an example of the LRS data and gait measurement results in a situation in which both legs were close together. As shown in Figure 9, in both data associations of Method 2 and the proposed method, the observed position of the right leg was disconnected from the validation region of the right leg at time *t* = 7.55.

In Method 2 with a large fixed validation region $\left(r_{val}^{f}=\frac{3}{2}w_{l}\right)$, the right validation region included the observed position of the left leg and the left validation region included the observed positions of the left and right leg. In this situation, false tracking by switching the left and right legs occurred with the GNN algorithm. To avoid switching of the legs in these situations, the validation region should be set smaller. However, as shown in Table 3, in Method 3 with a small fixed validation region ($r_{val}^{f}$ = *w_l_*), losing track of the leg is likely to occur when the velocity of the leg changes suddenly or the leg is moving while hidden from the sensor. In the proposed method with variable validation regions based on the state of each leg, the observed position of the right leg was disconnected from the small validation region of the left leg because the left leg was in the stance phase at time *t* = 7.55. The right validation region was expanded because the corresponding observed position did not exist within it, then the system detected the right leg at time *t* = 7.65.

As shown in Table 3, it was confirmed that the proposed leg detection and data association method can reduce the number of occurrences of lost tracking of legs and of false tracking.

**Table 3:** Leg tracking results for each method.

	Five Observed Leg Patterns	Radius of the Validation Region $r_{val}^{f}$	Participants not Using a Stick (14 people, 42 Trials)		Participants Using a Stick (2 People, 6 Trials)		Total (16 People, 48 Trials)
			Number of Lost Tracks	Number of False Tracks	Number of Lost Tracks	Number of False Tracks	Success Rate
Method 1	No	$\frac{3}{2}w_{l}$	6	5	3	0	70.8% (34/48)
Method 2	Yes	$\frac{3}{2}w_{l}$	1	1	0	2	91.7% (44/48)
Method 3	Yes	$w_{l}$	14	0	1	2	64.6% (31/44)
Proposed	Yes	Variable	0	0	0	2	95.8% (46/48)

### 3.3. Verification of Walking Parameters Extraction

To verify the validity of the target step judgment and cross step detection of the proposed system, we recorded performance on the MTST using video cameras and compared our results with those obtained using video analysis. Additionally, we verified the validity of the foot contact time and position using the results of the target step judgment.

**Figure 10:**
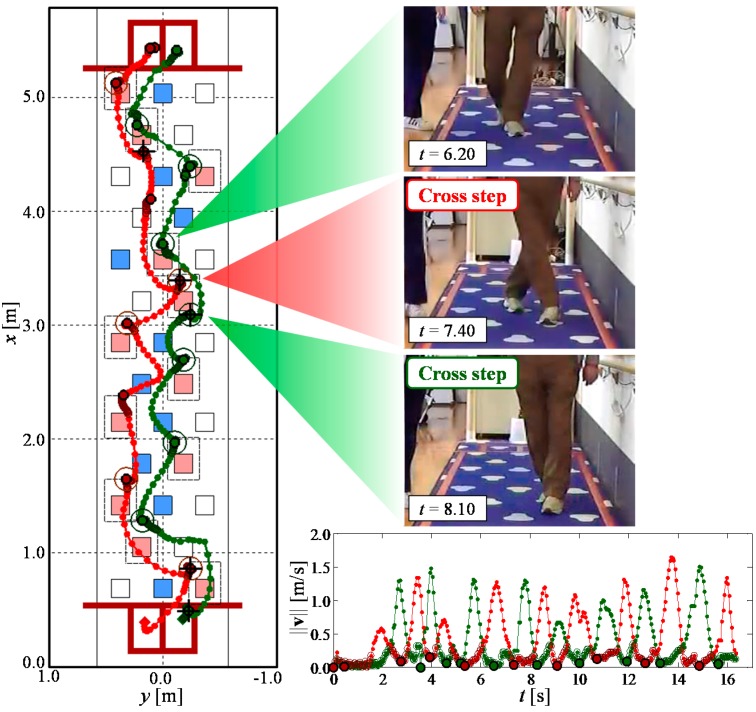
Example of gait measurement results.

Table 4 shows the results of target step judgment and cross step detection from 46 successful tracking data series compared with video analysis. Figure 10 shows an example of leg trajectory results. In Figure 10, if the system judged a target step, a large “O” symbol was displayed at the foot contact position. If the system detected a cross step, a large “+” symbol was displayed at the foot contact position. As shown in Figure 10 and Table 4, it was confirmed that the proposed system could judge target steps with very high accuracy (success rate: 99.0%), even including the participants using a stick. Additionally, the validity of the foot contact time and position obtained by the proposed system because of the high accuracy of the target step judgment was confirmed. We also confirmed that the proposed system could detect cross steps with high accuracy (success rate: 78.9%).

**Table 4:** Results of target step judgment and cross step detection.

	Participants not Using a Stick (381 Steps, 16 Cross Steps)		Participants Using a Stick (33 steps, Three Cross Steps)		Total (414 Steps, 19 Cross Steps)	
	Number of Non-Judgments and Non-Detections	Number of Misjudgments and False Detections	Number of Non-Judgments and Non-Detections	Number of Misjudgments and False Detections	Success Rate of Judgment and Detection	Rate of Misjudgment and False Detection
Target step judgment	4	3	0	3	99.0% (410/414)	1.4% (6/414)
Cross step detection	2	0	2	0	78.9% (15/19)	0.0% (0/15)

## 4. Conclusions

This study presents a gait measurement system using a LRS for the MTST to evaluate the risk of falling. The system is advantageous over current systems for the MTST in terms of cost, scale and convenience of use. When elderly people at high risk of falling perform the MTST, situations in which one leg is hidden from the sensor or the legs are close occur and are likely to lead to losing track of the legs or to false tracking. To solve these problems, we proposed a novel leg detection method with five observed leg patterns and GNN-based data association with a variable validation region based on the state of each leg. In addition, we proposed methods to judge whether the participant steps on the assigned colored targets (target step judgment) and detect a behavior where the swinging leg crosses against the supporting leg (cross step detection) based on the trajectory of both legs.

To verify the validity of the proposed gait measurement system, we carried out the MTST with 16 elderly people, including two elderly people using a stick. Comparing the experimental results with video analysis, we confirmed that the proposed system could improve leg-tracking performance, judge target steps and detect cross steps. We also confirmed the validity of the foot contact time and position obtained by the proposed system from the results of the target step judgment. This gait measurement system may be helpful in assessing fall risk indicators in the elderly in community health centers.
